# “I had so many life-changing decisions I had to make without support”: a qualitative analysis of women’s pregnant and postpartum experiences during the COVID-19 pandemic

**DOI:** 10.1186/s12884-022-04816-7

**Published:** 2022-07-04

**Authors:** Grayson B. Ashby, Kirsten A. Riggan, Lily Huang, Vanessa E. Torbenson, Margaret E. Long, Myra J. Wick, Megan A. Allyse, Enid Y. Rivera-Chiauzzi

**Affiliations:** 1 Mayo Clinic Alix School of Medicine, Rochester, MN USA; 2 Biomedical Ethics Research Program, Mayo Clinic, Rochester, MN USA; 3 Department of Quantitative Health Sciences, Mayo Clinic, Jacksonville, FL USA; 4 Department of Obstetrics & Gynecology, Mayo Clinic, Rochester, MN USA

**Keywords:** SARS-Cov-2, Pregnancy, Postpartum, Mental health, Qualitative research

## Abstract

**Background:**

The COVID-19 pandemic has posed profound challenges for pregnant patients and their families. Studies conducted early in the pandemic found that pregnant individuals reported increased mental health concerns in response to pandemic-related stress. Many obstetric practices changed their healthcare delivery models, further impacting the experiences of pregnant patients. We conducted a survey study to explore the ways in which COVID-19 impacted the lives of pregnant and newly postpartum people.

**Methods:**

A mixed-methods survey was distributed to all patients ≥18 years old who were pregnant between January 1st, 2020 – April 28, 2021 in a large Midwest health system. Open-ended survey responses were analyzed for common themes using standard qualitative methodology.

**Results:**

Among the 1182 survey respondents, 647 women provided an open-ended response. Of these, 77% were in the postpartum period. The majority of respondents identified as white, were partnered or married, and owned their own home. Respondents reported feeling greater uncertainty, social isolation, as though they had limited social and practical support, and negative mental health effects as a result of the pandemic. Many cited sudden or arbitrary changes to their medical care as a contributing factor. Though in the minority, some respondents also reported benefits from the changes to daily life, including perceived improvements to medical care, better work-life balance, and opportunities for new perspectives.

**Conclusions:**

This large qualitative dataset provides insight into how healthcare policy and lifestyle changes impacted pregnant and postpartum people. Respondents expressed similar levels of uncertainty and mental health concerns compared to other cohorts but less overall positivity. Our findings suggest greater attention be given to the impact of pandemic-related stress on pregnant and postpartum women. As the pandemic continues, these data identify areas where investment in additional support may have the greatest impact.

## Background

On March 16, 2020, the World Health Organization declared the novel SARS-Cov-2 coronavirus (COVID-19) outbreak a pandemic [1]. COVID-19 has profoundly impacted all aspects of human society. In addition to significant morbidity and mortality as a direct result of COVD-19 infection, the changes to everyday life initiated to prevent transmission have caused downstream socioeconomic effects, including loss of income, social isolation, and anxiety [2–4]. Pregnant and postpartum women have been particularly impacted by dramatic changes to their pregnancy and birth experience because of healthcare measures to conserve resources and restrict COVID-19 transmission. Although each health system initiated their own policies, common changes included transition to telehealth or drive-thru prenatal appointments; bans on visitors and/or partners during prenatal procedures and delivery; and increased planned home deliveries to avoid potential COVID-19 hospital exposures [5–7]. Pregnancy is now known to be a comorbidity for severe COVID-19 disease; pregnant women who contracted SARS-CoV2 and are symptomatic have a two-fold increased risk of requiring ICU admission, invasive ventilation, and extracorporeal membrane oxygenation (ECMO); a 70% increased risk of death; and increased risk of perinatal complications [8–12].

Early in the pandemic, pregnant and postpartum women reported increased depressive and anxiety symptoms, which may be related to structural changes in daily life coupled with concerns about contracting the disease [13–15]. The perinatal period is associated with increased risk for mood disorders, and events like the current pandemic exacerbate maternal mental health concerns [16, 17]. Mental health disorders are also associated with increased risk for psychiatric, developmental, and other chronic disorders in children that can lead to long-term morbidity [18–22].

The aim of our study was to characterize the impact of the COVID-19 pandemic on pregnant and postpartum women in a large Midwestern health system by exploring their experiences with prenatal care and support in the perinatal period.

## Methods

### Survey development

We utilized the Coronavirus Perinatal Experiences Impact Survey (COPE-IS), a 90-item, mixed-method open-source survey designed by experts in maternal health to collect data on the effect of the COVID-19 pandemic on perinatal experiences [23]. The COPE-IS survey has been broadly employed throughout localities in the United States and Europe for future cross-sectional data analysis [24–26], but has not been psychometrically tested because of the novelty of the pandemic. Respondents are asked to answer single-answer, multiple-choice, and Likert Scale questions regarding pregnancy and postpartum concerns, feelings of support, and challenges due to the pandemic. Demographic information is also collected. Respondents are also asked to answer open-ended questions about positive and negative life changes due to the pandemic, sources of stress, and advice they would give to others in similar situations.

### Recruitment and data collection

Mayo Clinic’s Institutional Review Board determined this study to be exempt in accordance with 45 CFR 46.104d, category 2 as only anonymous survey data was collected. Eligible patients were determined using Mayo Clinic’s electronic health record database. To be included in this study, participants met the following criteria: pregnant as of January 1st, 2020 – April 28, 2021, English-comprehending, and age ≥ 18 years old. Participants were excluded if they were pregnant outside of the timeframe, under age 18, had a miscarriage or stillbirth during this time period or did not have an email address on file. Participants who met inclusion criteria received an invitation to complete the anonymous survey created through REDCap (REDCap 11.1.20 –© Vanderbilt University. Nashville, TN), a secure web-based application for data collection. Two reminder emails were sent following the initial email. The survey was open from April 28, 2021 – June 28, 2021.

### Analysis

Quantitative findings are reported separately. All open-ended responses were included for qualitative analysis. NVivo 12 (QSR International, Melbourne, Australia), a qualitative data analysis software, was used to facilitate coding open-ended questions according to standard qualitative methodology [27]. Thematic qualitative analysis is a common analytic approach for understanding emerging phenomena. The advantages of this approach include the ability to characterize novel pregnancy and postpartum experiences in-depth, including unanticipated or more nuanced experiences and insights that may not be captured in fixed-choice responses. This is an essential component of the study design given the unprecedented nature of the COVID-19 pandemic.

Two researchers (GBA and LH) created a codebook based on iteratively identified topics after initial review of responses. The codebook was validated via consensus coding between two researchers (GBA and LH) for the first 50 responses (7.2%) with additional refinement of the codebook throughout the coding process. The final codebook was applied to all responses. The remaining responses were divided evenly between the two researchers, with intermittent consensus coding totaling 150 (21.7%) of the responses to maintain coding accuracy. The two reviewers also met weekly to clarify any discrepancies that emerged during the coding process. In accordance with the Standards for Reporting Qualitative Research, direct quotes are reported [28]. Quotes have been minimally edited for readability.

## Results

### Demographics

Out of 6141 survey invitations, 1182 respondents completed the survey (19.1% response rate), with 647 respondents answered at least one open-ended question. Demographics are provided in Table 1. Three major themes were identified: heightened uncertainty around social and medical safety, the experience of social isolation during pregnancy, birth, and neonatal care, and negative mental health. A minority of respondents indicated that some COVID-19 prevention measures resulted in positive changes to daily life and medical care.

**Table 1:** Participant demographics

Characteristic	Pregnant (***N*** = 149)	Postpartum (***N*** = 498)	Total (***N*** = 647)
**Age (years)**	*N* = 120	*N* = 435	*N* = 555
Mean (SD)	32.6 (3.7)	32.7 (4.4)	32.7 (4.2)
Range	(22.3–43.4)	(21.2–52.2)	(21.2–52.2)
**Education**	*N* = 147	*N* = 496	*N* = 643
Less than 12th Grade	1 (0.7%)	1 (0.2%)	2 (0.3%)
High School Degree/GED	7 (4.8%)	23 (4.6%)	30 (4.7%)
Trade school/apprenticeship	0 (0.0%)	6 (1.2%)	6 (0.9%)
Partial College	11 (7.5%)	38 (7.7%)	49 (7.6%)
2-year College Degree	11 (7.5%)	54 (10.9%)	65 (10.1%)
4-year College Degree	69 (46.9%)	218 (44.0%)	287 (44.6%)
Graduate Degree	48 (32.7%)	156 (31.5%)	204 (31.7%)
**Relationship status**			
Single	6 (4.0%)	14 (2.8%)	20 (3.1%)
Partnered/Married	141 (94.6%)	475 (95.4%)	616 (95.2%)
Divorced/Separated	0 (0.0%)	8 (1.6%)	8 (1.2%)
Other	2 (1.3%)	1 (0.2%)	3 (0.5%)
**Race**	*N* = 146	*N* = 473	*N* = 619
Black or African American	1 (0.7%)	2 (0.4%)	3 (0.5%)
Native American/ Alaska Native	1 (0.7%)	3 (0.6%)	4 (0.6%)
Native Hawaiian/ Pacific Islander	0 (0.0%)	1 (0.2%)	1 (0.2%)
Asian	3 (2.1%)	9 (1.9%)	12 (1.9%)
White	136 (93.2%)	448 (94.7%)	584 (94.3%)
Other	1 (0.7%)	1 (0.2%)	2 (0.3%)
Mixed	4 (2.7%)	9 (1.9%)	13 (2.1%)
**Ethnicity**	*N* = 137	*N* = 455	*N* = 592
Hispanic or Latino	2 (1.5%)	12 (2.6%)	14 (2.4%)
Not Hispanic or Latino	135 (98.5%)	443 (97.4%)	578 (97.6%)
**Household income in the past year**	*N* = 145	*N* = 485	*N* = 630
Less than $40,000	7 (4.8%)	39 (8.0%)	46 (7.3%)
$40,000 to $60,000	16 (11.0%)	54 (11.1%)	70 (11.1%)
$60,000 to $80,000	22 (15.2%)	68 (14.0%)	90 (14.3%)
$80,000 to $100,000	22 (15.2%)	82 (16.9%)	104 (16.5%)
$100,000 to $120,000	26 (17.9%)	77 (15.9%)	103 (16.3%)
$120,000 to $160,000	29 (20.0%)	85 (17.5%)	114 (18.1%)
$160,000 to $200,000	8 (5.5%)	30 (6.2%)	38 (6.0%)
$200,000 to $250,000	8 (5.5%)	17 (3.5%)	25 (4.0%)
Greater than $250,000	7 (4.8%)	33 (6.8%)	40 (6.3%)
**Do you currently cohabitate with a partner?**			
Missing	1	2	3
No	3 (2.0%)	15 (3.0%)	18 (2.8%)
Yes	145 (98.0%)	481 (97.0%)	626 (97.2%)
**How long have you lived with this partner?**			
Missing	4	17	21
Less than 1 year	0 (0.0%)	1 (0.2%)	1 (0.2%)
1–3 years	12 (8.3%)	48 (10.0%)	60 (9.6%)
3–6 years	51 (35.2%)	126 (26.2%)	177 (28.3%)
6–9 years	36 (24.8%)	142 (29.5%)	178 (28.4%)
9 or more years	46 (31.7%)	164 (34.1%)	210 (33.5%)
**Which best describes where you currently live:**			
Missing	1	0	1
A 1 bedroom dwelling	1 (0.7%)	5 (1.0%)	6 (0.9%)
A multi-bedroom dwelling	147 (99.3%)	492 (98.8%)	639 (98.9%)
I do not have a stable housing arrangement	0 (0.0%)	1 (0.2%)	1 (0.2%)
**Has your living environment changed since the beginning of the pandemic?**			
Missing	1	0	1
No	119 (80.4%)	414 (83.1%)	533 (82.5%)
Yes	29 (19.6%)	84 (16.9%)	113 (17.5%)
**Has the change to your living environment had a positive or negative impact?**			
Missing	120	414	534
Very positive	14 (48.3%)	31 (36.9%)	45 (39.8%)
Somewhat positive	8 (27.6%)	26 (31.0%)	34 (30.1%)
Somewhat Negative	1 (3.4%)	18 (21.4%)	19 (16.8%)
Very Negative	2 (6.9%)	6 (7.1%)	8 (7.1%)
No Impact	4 (13.8%)	3 (3.6%)	7 (6.2%)
**Do you currently own or rent your residence?**			
Missing	1	0	1
Owned by you or someone in the household	133 (89.9%)	434 (87.1%)	567 (87.8%)
Rented	12 (8.1%)	59 (11.8%)	71 (11.0%)
Occupied without payment of rent	0 (0.0%)	1 (0.2%)	1 (0.2%)
Temporarily living with others	2 (1.4%)	3 (0.6%)	5 (0.8%)
Other	1 (0.7%)	0 (0.0%)	1 (0.2%)
I decline to answer	0 (0.0%)	1 (0.2%)	1 (0.2%)
**How many people currently live in your home (including self)?**			
Missing	0	1	1
2	4 (2.7%)	6 (1.2%)	10 (1.5%)
3	79 (53.0%)	166 (33.4%)	245 (37.9%)
4	41 (27.5%)	183 (36.8%)	224 (34.7%)
5	17 (11.4%)	83 (16.7%)	100 (15.5%)
6	6 (4.0%)	38 (7.6%)	44 (6.8%)
7	2 (1.3%)	13 (2.6%)	15 (2.3%)
8	0 (0.0%)	2 (0.4%)	2 (0.3%)
9	0 (0.0%)	2 (0.4%)	2 (0.3%)
10	0 (0.0%)	3 (0.6%)	3 (0.5%)
More than 15	0 (0.0%)	1 (0.2%)	1 (0.2%)
**Number of children:**			
Missing	4	1	5
1	86 (59.3%)	185 (37.2%)	271 (42.2%)
2	37 (25.5%)	179 (36.0%)	216 (33.6%)
3	15 (10.3%)	83 (16.7%)	98 (15.3%)
4	6 (4.1%)	35 (7.0%)	41 (6.4%)
5	1 (0.7%)	11 (2.2%)	12 (1.9%)
6	0 (0.0%)	2 (0.4%)	2 (0.3%)
8	0 (0.0%)	2 (0.4%)	2 (0.3%)
**Number of adults:**			
Missing	1	5	6
1	2 (1.4%)	8 (1.6%)	10 (1.6%)
2	131 (88.5%)	452 (91.7%)	583 (91.0%)
3	14 (9.5%)	19 (3.9%)	33 (5.1%)
4	1 (0.7%)	9 (1.8%)	10 (1.6%)
5	0 (0.0%)	5 (1.0%)	5 (0.8%)

### Uncertainty

Many respondents described long periods of significant uncertainty during the COVID-19 pandemic. Unforeseen changes to their medical care at all stages of their pregnancy and postpartum period were a major source of this uncertainty. Most women reported frustration or disappointment with the medical care they received. Key hospital policies that were frequent sources of frustration included limitations to the number of support people during labor and delivery, restrictions on hospital re-entry if the support person left the hospital, and inability to walk in the hallways while laboring, all of which changed at different stages in the pandemic. An approximate timeline of hospital policy changes initiated by our hospital system in response to the pandemic can be seen in Table 2.

“Given my birth was early on in the pandemic, not much was yet known so things changed each week. For my planned induction I was confined to the room and unable to walk to help with labor. My husband was not able to come and go as planned in order to take care of our other children.” (Respondent 27, Age 38, Delivered May 2020).

Others felt that the masking policy disrupted their labor and postpartum recovery. This hospital policy was changed after testing on admission became readily available.

“The birth was weird. I labored for 36 hours, [and] most of that time I had to wear a mask even though I had tested negative for COVID-19 2 days prior. I found it somewhat hard to bond with our new baby because of the masking. This was the hardest and most different birthing process I have had.” (Respondent 65, Age 36, Delivered July 2020).

For many, feelings of uncertainty stemmed from general concerns about COVID-19 morbidity and mortality. However, many women also reported specific concerns over a lack of knowledge of how COVID-19 and pregnancy and long-term impacts for their children.

“[I’m concerned with the] risk of baby and myself contracting COVID and how that could affect my baby’s health.” (Respondent 431, Age not disclosed, Delivered August 2021).

Childcare was also a source of uncertainty. Many families faced interruptions to their normal routines when childcare centers shut down either full time or intermittently in the event of an exposure. Given uncertainty around symptoms and the effect of COVID-19, many children also had to remain out of childcare if they experienced otherwise benign respiratory symptoms.

“The negative impacts and conflicting guidelines for daycare meant our children were out for many days in a row even if it was simply a runny nose from allergies without any other symptoms including fevers. Eventually I was frustrated and sick enough of this … so I did ask my employers to go to 75% time with benefits to allow me more time at home while they're young.” (Respondent 433, Age 31, Delivered January 2020).

Others experienced employment difficulties as a result of companies adapting to the pandemic, including lost wages and/or reduced hours.

“The additional stressor of having to take a pay cut and possible furlough only a couple of months before giving birth was also incredibly scary.” (Respondent 396, Age 33, Delivered July 2020).

“My longtime employer of 12 years decided not to honor a leave of absence, so I currently have lost my career. After working full-time for close to two decades, I was not expecting this change.” (Respondent 329, Age 39, Delivered March 2020).

Many respondents who reported currently or previously working in the healthcare sector described increasing demands related to the pandemic combined with uncertainty of the impact of a COVID-19 infection on themselves and their unborn children.

“I was working in the COVID ICU while pregnant and did not feel that I had adequate support or resources from supervisors/management to help me navigate the difficult situation I was in.” (Respondent 488, Age 30, Delivered, August 2020).

**Table 2:** Timeline of hospital policies

	Pre-COVID-19	Post-COVID-19
**Masks**	No masking	Required for patients, visitors, and staff in all areas of hospital
**Prenatal/Postnatal Care**	In-person	In-person and virtual options
**Inpatient visitors**	No limitations on number, age, or hours	Initially 1 consistent visitor > 16 years old with no hospital re-entry allowed Later transitioned to 2 consistent visitors > 5 years old with re-entry allowed
**COVID positive women**		Initially no visitors and infant stays with mother unless in NICU Later transitioned to allow approved visitation with appropriate PPE
**Hallway access for walks during inpatient stay**	Unrestricted	Unavailable for all initially, but later unrestricted if COVID test negative
**NICU visitation**	Limited to 2 consistent individuals during flu seasons	Initially 1 consistent visitor, but later 2 consistent visitors

### Social isolation and socialization

Another prominent theme was the impact on social interaction and infant socialization due to social distancing, stay-at-home orders, and the cancellation of most group activities.

“The hardest part of the pandemic has been the social isolation from family while I was pregnant and then with a new baby (born June 2020). Most of my family is missing his first year of life and so much change happens during that first year.” (Respondent 445, Age 33, Delivered June 2020).

Several respondents reported feeling that they were missing a more traditional pregnancy experience, including baby showers and postpartum visitors. They were also concerned that their extended families missed out on seeing their baby.

“We missed out on so much … birthing classes, breastfeeding classes/support … no baby shower, no diaper party, moved without family and friends. The list goes on and on … The hospital staff didn’t even offer to take a picture of us as a family in the hospital so now we will never have one.” (Respondent 453, Age 32, Delivered May 2020).

Other respondents felt that limited social interactions will hamper future social development of their infant, as well asl impair immune system function due to reduced exposure to childhood illnesses.

“[I’m] concerned that masked faces may potentially inhibit development of babies and young children, as not developing facial recognition, seeing people smile at them, etc.” (Respondent 123, Age 44, Delivered July 2020).

“I am worried that my baby’s lack of socialization will negatively impact him not only socially, but that he has not been exposed to the normal microbes that most children are and he may not have built up immunity, therefore being more prone to illnesses later in life.” (Respondent 418, Age 31, Delivered June 2020).

### Mental health

In addition to physical isolation, respondents reported that COVID-19 related restrictions placed additional mental health burdens on pregnant women who were often prevented from seeking traditional forms of emotional and practical support during and after pregnancy.

“My husband couldn’t come to any prenatal ultrasounds and appointments even though I had a high-risk pregnancy. It was awful not having any support person in those moments.” (Respondent 1082, Age 30, Delivered April 2020).

“When my son was a newborn, I struggled with sleep deprivation. It was very, very hard not to be able to call someone to come over and watch the baby for a few hours while I slept. This was profoundly difficult on my mental health.” (Respondent 203, Age 40, Delivered July 2020).

Lack of support often led to perceived feelings of stress and self-reported anxiety and depression, prompting some to seek care for their mental health concerns. In some cases, reduced healthcare visits led to a delay in symptom recognition and diagnosis.

“My partner has been struggling and gone multiple days at a time. It puts a lot of stress on me. I am raising 4 children as a single mom, without childcare, most of my support people are gone and I work full-time.” (Respondent 223, Age 35, Delivered August 2020).

“COVID restrictions greatly reduced my follow-up cares, and I was not brought back for a routine 6-week postpartum follow-up. As a result, I think I struggled with postpartum depression for 2-3 months before recognizing my symptoms and getting help.” (Respondent 392, Age 33, Delivered March 2020).

### Positive reflections

While almost all respondents reported some form of negative impact of the pandemic, some also described positive pandemic-related changes to their medical care, including remote healthcare options and abbreviated hospital stays post-delivery, although positive changes were less reported than negative impacts. Among these, several women were supportive of shortened hospital stays because this placed less childcare pressure on already strained support networks.

“Telehealth and 24-hour hospital stay were awesome - loved every minute of it.” (Respondent 200, Age 36, Delivered June 2020).

Respondents also recounted unforeseen benefits, such as decreased pressure to socialize, more time and flexibility, and greater opportunities for self-care.

“Normally my older two would be in preschool and kindergarten, but I got to spend all day everyday with them. We made a lot of positive changes in our lives - stopped eating out, more family time with those close to us, more exercise at home.” (Respondent 69, Age 36, Delivered February 2020).

“Less pressure to be involved with many activities - I liked the slower pace of life. I liked NOT having visitors during my post-partum stay. Much more restful and private.” (Respondent 179, Age 41, Delivered July 2020).

Some respondents also voiced positive changes at work like transitioning to remote work.

“My work has realized that I can do my job remotely, which has given me 2 hours of my day back which I am able to spend with my children. Without this drive time I was able to shorten up daycare hours and cost.” (Respondent 27, Age 38, Delivered May 2020).

Lastly, some women reflected on gratitude, empathy, and resilience in response to the COVID-19 pandemic.

“I have less anxiety [now that] I've seen how if plans are canceled and ruined and support systems (schools/ churches/government) are not available, my family and I are still okay. We can survive tough things.” (Respondent 899, Age 33, Delivered May 2021).

## Discussion

The purpose of this study was to understand the experiences of pregnant and postpartum women during the COVID-19 pandemic in an upper Midwest cohort. Specific themes that arose in our population included heightened uncertainty around medical care and pandemic-related changes, social isolation, and negative mental health impacts. However, a minority of respondents found moments of positivity in the pandemic, including more time and flexibility for activities like self-care or family bonding, a slower pace of life, and gratitude for their circumstances despite the surrounding chaos and uncertainty. Much of the initial literature around COVID-19 and the perinatal period focused on triage and the provision of medical care, but more recently published data have shed light on how the COVID-19 pandemic influenced other aspects of care that are important to our patients.

To our knowledge, Kinser et al. have been the only group to report qualitative data from pregnant and postpartum women using the COPE-IS survey [24]. They surveyed this population in April–June 2020 at the height of the initial shutdown in an urban Southeastern US health system. Their qualitative analysis similarly revealed significant fear, anxiety, and mental health difficulties from the pandemic, though they reported more concerns over supply shortages, unemployment, and health insurance than we found in our population. These differing responses may reflect the stage of the pandemic during which the survey was distributed. However, both populations expressed fears of illness and death from COVID-19 – not only for themselves but also for loved ones. Both populations reported restricted social support during all stages of pregnancy and the postpartum period as well as sadness over the loss of an otherwise “normal” pregnancy experience. Interestingly, in Kinser and colleagues’ population, the majority of respondents reported gratitude for a change in perspective, including more time with family, opportunities for self-care, and a slower pace of life, whereas in our population, such feelings were reported by a minority of women.

Others have also reported qualitative data about the impact of COVID-19 on prenatal care using different survey instruments or via semi-structured interviews [29–32]. Javid et al. identified similar structural changes in healthcare and behavioral changes to reduce disease transmission (e.g. virtual visits and absence of support people, respectively) that led to emotional responses of increased fear, anxiety, and feeling unsupported. Others reported similar levels of both generalized uncertainty around the pandemic and perinatal care and more specific concerns related to the effects of viral infection on their own health and their children, feelings of missing out on a more traditional pregnancy experience, and challenges from limited family and medical care support [30–32].

Our data, in conjunction with the reports from other cohorts, suggest that during the COVID-19 pandemic, pregnant women in the United States felt reduced levels of support during their pregnancy as they navigated the changes to pregnancy care and delivery during a pandemic. However, additional data are needed from certain subpopulations, including single women, urban populations, and regions hit hardest by the pandemic, to gain a comprehensive picture of the similarities or differences based on population-level factors.

### Sources of uncertainty

Many women experience feelings of uncertainty during pregnancy in non-pandemic circumstances [33]. Our results suggest that the onset of COVID-19 resulted in two inter-related sources of increased uncertainty experienced by pregnant and recently pregnant women during this time. The first was the constant shifting of policies enacted by healthcare systems to adjust to the changing state of the pandemic. These policies included alternative models for prenatal care and various infection prevention measures for patients and staff. Within our own health system, our policies on visitation, masking, and admission testing changed from week to week based on government recommendations, risk of community spread, and site-specific factors.

The second, related source of uncertainty was the sudden onset of the pandemic and the rapidly shifting knowledge about infectivity, mode of transmission, morbidity, and effective prevention strategies. In particular, data on COVID-19 infection in pregnant women, infants, and children lagged significantly behind other demographic groups. One of the earliest reports describing the effects of COVID-19 on pregnant women was not released until mid-June 2020–3 months after the WHO declaration – and much of the data for neonates and infants was not reported until Summer 2021 [34–36]. This left pregnant women without the knowledge to make informed decisions about their care and prevention strategies, likely adding to their distress.

Prior to COVID-19, women have said that some of the inherent uncertainty of pregnancy fades with each successive pregnancy, but in our cohort, feelings of uncertainty did not appear mediated by parous status, with several respondents reporting that the pandemic birth experience was more stressful than their previous delivery experiences [37]. In a state of unprecedented uncertainty, the healthcare system enacted policies to reduce disease transmission and enhance the safety of prenatal and delivery care in a fluid environment; unfortunately, many in our population found these policies frustrating and disruptive during a pivotal life experience. Stress exposure in pregnancy and the postpartum period, however, are known risk factors for poor maternal mental health and adverse health outcomes in children [18–20, 22, 38–42]. While important to acknowledge the role that new and shifting policies played in heightening uncertainty, we also recognize these changes were enacted by healthcare institutions out of the utmost concern for patient health and safety. A recent study of OBGYN providers during the COVID-19 pandemic showed they were also experiencing significant frustration and anxiety with changing policies [43]. As healthcare systems respond to new SARS-Cov-2 variants of concern, they should integrate the knowledge gained from this dataset, and others, into their policymaking decisions to mitigate the stress experienced by pregnant and postpartum women. Improved communication of policy changes and evidence supporting such changes may promote transparency and mitigate feelings of uncertainty around pregnancy care and the delivery experience.

Though many women found these policies restrictive or burdensome, some women reported an improved pregnancy and delivery experience. These respondents appreciated the flexibility offered by telehealth, although some expressed concern that pregnancy and postpartum complications would be missed without a physical exam. Other pandemic surveys also demonstrated that both patients and prenatal care providers found virtual prenatal care to be feasible and satisfactory, although patients reported a strong preference for in-person appointments if given the choice [44, 45]. Since 2014, our health system has researched and implemented a reduced-frequency prenatal care model enhanced with remote home monitoring devices and nursing support [39]. As such, many of our healthcare teams quickly adapted to this model, which may have contributed to some of the positive experiences seen in our respondents. Some serendipitous improvements could serve as launch changes to current perinatal care patterns, with future research looking at not just maternal and fetal health outcomes but also psychosocial considerations.

### Loss of support and mental health

An additional source of considerable stress was the impact of changing policies on society more generally, including limitations on groups and social interaction, closures of schools and childcare centers, and reduced employment. The basal stress that accompanies pregnancy, birth, and caring for a newborn was layered onto the societal impact felt by everyone.

Perceptions of limited social support also affected this population. Pregnancy and the postpartum period carry a known risk of increased mental health concerns, and previous studies have demonstrated that social support is a protective factor against developing postpartum depression [20, 40]. Traditional avenues for assistance or respite care, such as older grandparents, family members, or friends, were limited due to social distancing and the increased COVID-19 risk among older populations [46]. Those at even higher risk for isolation include single parents or others with limited support networks prior to the pandemic. Many respondents described additional responsibilities when daycare centers and schools closed indefinitely, juggling distance learning and care of a newborn alongside responsibilities at home and in the workplace. A qualitative interview study of young parents from the United Kingdom similarly pointed to limited social support as well as economic instability as key pandemic stressors that negatively influenced mental health in a population already at-risk for mental health concerns [32].

A minority of respondents felt pandemic changes had a positive effect on their mental well-being. Infection mitigation plans produced an environment where families could have more time at home, with fewer parents leaving the house for work and children for school or daycare, and fewer social obligations, which is supported by other qualitative findings on postpartum experiences during the COVID-19 pandemic [30]. Some expressed relief in not having to entertain visitors during the postpartum period. Though some thrived in a newfound environment of self-care, others found less margin for respite or time alone, which has been associated with increased depressive symptoms during the postpartum period [47]. Very few mentioned adopting new behaviors to protect their mental health, such as decreased use of social media or limiting news consumption.

A systematic review by Harville et al. demonstrated that exposure to disasters (e.g. terrorist attack, earthquake) increases risk for mental health issues for pregnant and postpartum women, and these data provide qualitative accounts that support their findings [17]. Increased support of pregnant and postpartum women should be prioritized in times of need or social unrest given that isolation is a known risk factor for adverse maternal-fetal outcomes, including preterm birth and low birth weight [48, 49]. Further, postpartum depression also increases risk for maternal risk factors like substance abuse, suicidal ideation, and poor physical or mental health, or that ultimately impacts infant development [50]. While beyond the scope of this study, maternal mental and physical health are also important for childhood development [51]. This further highlights the importance of ensuring robust support for women and children during the perinatal period as well as long-term medical and psychosocial care for any children born during the pandemic period who may be impacted by heightened maternal stress and adverse mental and physical health.

### Strengths and limitations

Strengths of this study include a large sample size for a qualitative dataset and detailed responses to the open-ended survey items, reflecting a variety of experiences. This survey was administered in Spring 2021 after medical knowledge and response to the COVID-19 pandemic was more solidified, with widely available testing and vaccines released to adults in the general public, so our results may not reflect the entire pandemic experience. The temporal distance from birth and prenatal care for women who delivered earlier in the pandemic may lead to recall bias. The low survey response rate (19%) is also a limitation; while respondent experiences are believed to be representative of this population, non-response bias may also be a factor. Generalizability of these findings is limited because of the regional nature of pandemic surges and varying local responses, including hospital policy changes, and limited racial/ethnic diversity. Though representative of the survey catchment area, the study findings do not capture the experiences of patients of color who are known to be at higher risk for poor maternal and fetal outcomes and mental health problems and who may experience disproportionate burdens of the pandemic. As the survey was delivered by email only, populations with limited or no internet access and those with limited English proficiency were not represented. Lastly, we distributed this survey over a year after COVID-19 had been declared a pandemic, which may have allowed time for reflection and influenced their ability to report positive pandemic-related changes that may have initially been reported negatively. Pandemic fatigue may have resulted in more negative responses. Additional research is needed to understand how pregnant and postpartum women are facing evolving challenges from the COVID-19 pandemic, such as how pregnant and breastfeeding women approach vaccination for themselves and their children, navigating daycare and school reopening or playdates with the continued risk of COVID-19 transmission, and how these women interact with the healthcare system after the designation of COVID-19 as a pandemic is removed. Further, because the COPE-IS survey was designed for pregnant and postpartum women, fathers were not included in this survey, but understanding how the COVID-19 pandemic impacted fathers or partners, including restrictions on support persons in labor and delivery, is another avenue for further research.

## Conclusions

Pregnant and postpartum women face unique challenges during the ongoing COVID-19 pandemic; this study identified increased uncertainty, social isolation, and lack of support as potential drivers behind the increased stress and mental health concerns seen in this population. Those caring for pregnant and postpartum women should be mindful of these concerns, and these data should inform future policy decisions among healthcare systems. As the current COVID-19 pandemic continues to affect pregnant and postpartum women, support in these areas may have the greatest impact.

## Data Availability

The dataset used during the current study is available from the corresponding author on reasonable request.
